# Co-design of a nurse handover tool to optimise infection control and antimicrobial stewardship in a low resource setting intensive care unit: A nurse led collaboration

**DOI:** 10.12688/wellcomeopenres.22931.1

**Published:** 2024-10-15

**Authors:** Candice Bonaconsa, Dena van den Bergh, Esmita Charani, Thouwybah Phillips, Aletta Spogter, Aghmat Mohamed, Dawood Peters, Ivan Joubert, Marc Mendelson

**Affiliations:** 1 Division of Infectious Diseases and HIV Medicine, Department of Medicine, University of Cape Town, Cape Town, Western Cape, 7925, South Africa; 2 Faculty of Health and Life Sciences, University of Liverpool, Liverpool, England, UK; 3 Division of Nursing, Intensive Care, Groote Schuur Hospital, Observatory, Western Cape, South Africa; 4 Division of Nursing Management, Groote Schuur Hospital, Observatory, Western Cape, South Africa; 5 Division of Critical Care, University of Cape Town Department of Anaesthesia and Perioperative Medicine, Cape Town, Western Cape, South Africa

**Keywords:** Nursing handover, Co-design, Infection control, Graphic facilitation, Antimicrobial stewardship, Participatory action research, Intensive care unit.

## Abstract

**Background:**

The quality of intensive care unit (ICU) nursing handover impacts patient safety, including infection prevention and control (IPC) and antimicrobial stewardship (AMS) practices. We report a co-designed quality improvement study using a visual, structured nurse handover tool in a low resource setting.

**Methods:**

The study was conducted with clinical nurses in an 8-bed medical ICU at a tertiary hospital in South Africa. Using a participatory action research (PAR) framework and visual participatory methods, the handover tool development had three phases: data collection, journal club, and co-design. To engage busy nurses and create real-time discussions and input, 7-minute focussed sessions in the ICUs using large-scale graphics to facilitate were used. Qualitative data were thematically analysed.

**Results:**

Between September – October 2022, baseline data were collected from 16 handovers (46 patient discussions over 4 hours). The tool was co-designed through 18 contact sessions involving 31 nurses (April–June 2023). Variation was observed in patterns of handover structure (sequence of what was presented) and content (type and the level of detail of information provided). An evidence-based visual tool was co-designed to identify and manage key patient care risk factors. The tool included a structured section to report on IPC and AMS. Nurses reported the visual prompts to be beneficial to ensuring consistent inclusion of critical information in handovers.

**Conclusions:**

An innovative approach involving ICU nurses in co-designing a visual handover tool resulted in a structured method for systematically reporting patient care risk factors, body systems, IPC, and AMS. Implementation and dissemination in this unit, and expansion to other units, is underway to promote sustainable change in nursing clinical practices.

## Plain language summary

Effective communication during handovers among healthcare workers (HCWs) is vital for providing safe, high-quality care to critically ill patients. Poor handover communication can lead to patient safety problems, such as delayed or incorrect treatments. Infection prevention and control (IPC) and antimicrobial stewardship (AMS) are vital in reducing drug-resistant infections in hospitals, especially in low and middle-income countries. Nurses, who play a key role in infection management, benefit from structured handover practices that ensure consistent and accurate information transfer.

In our study at a tertiary hospital in South Africa, we worked with 31 nurses to co-design a visual handover tool. We observed current handover practices, engaged nurses in an interactive journal club, and developed the tool through participatory action research. We used short, focused sessions, or "seven-minute scrums," and visual methods, including large scale graphics, to involve nurses from all categories despite their busy schedules. Leadership support was essential, with leaders showing openness to new approaches and encouraging nurse participation.

The visual tool, co-designed in short sessions, uses simple icons to ensure no information is lost, guiding a standardised and structured reporting on patient care and infection prevention. By actively involving nurses in the design process, we invited shared ownership and responsibility. This tool aims to enhance handover accuracy to improve patient care delivery in a low resource setting.

## Introduction

Effective healthcare worker (HCW) communication is crucial for delivering safe, high-quality, and uninterrupted care for critically ill patients ^1, 2^. Poor handover communication directly impacts patient safety, contributing to preventable adverse events in hospitalised patients ^3^. A lack of a standardised handover systems exacerbates communication failures within the ICU ^4^. Inaccurate or missing information during the nursing handover can lead to delayed or inappropriate treatment, patient harm, prolonged hospitalisation, and even death ^1, 3, 5^.

Infection prevention and control (IPC) and antimicrobial stewardship (AMS) are important interventions mitigating the spread of drug-resistant infections in hospitals ^6^. ICU patients, especially in low and middle-income countries (LMICs) face heightened infection risks ^7^. Nurses play a vital role in infection management, from recognising symptoms to timely and appropriate specimen collection and antibiotic administration ^8– 13^. Optimising HCW communication can positively impact infection-related patient outcomes ^14^. Structured communication during handovers, including IPC and AMS, facilitate proactive infection care. Consistent reporting between shifts ensures continuity of care, alongside with other patient priorities.

Co-design in health research involves collaboration between researchers, HCWs, and/or patients to develop, implement, and evaluate real-world solutions ^15^. Most of the existing successful co-design evidence is from high income countries, with limited data available from LMICs ^15^. Collaborative and inclusive methods, involving end-users to develop practical, context-specific solutions, leads to more sustainable outcomes in healthcare ^15– 19^. Co-design is complex due to existing hierarchies and working practices within healthcare teams ^15^. A recent study examined the impact of creative co-design approaches across 14 healthcare projects in the United Kingdom on service improvement and knowledge mobilisation. Interviews with participating clinicians and academics found that fostering creativity and establishing the right conditions for knowledge sharing, enhances participation and creates a safe, hierarchy-free environment ^19^.

Visually engaging interventions targeting patients and healthcare teams have shown to positively influence behaviour in healthcare settings ^20– 23^. Graphic facilitation, using simply drawn visuals and icons on large scale graphics, guides individuals towards a shared goal ^22, 24– 28^. For example, one study used visualisation methods to help focus-groups describe nursing practice involving families in the care of hospitalised children ^29^. Another study employed graphic facilitation to engage residents from a multi-ethnic, disadvantaged neighbourhood in Denmark to co-design and evaluate a health promotion intervention ^28^.

Our study involved ICU nurses in a participatory approach, leveraging the researcher's expertise in visualisation techniques to design a visual ICU nursing handover tool ^22, 23, 29– 31^. We identified current ICU handover practices and collaboratively designed and piloted the tool. We detail our co-design process, highlighting lessons learned and innovative practices developed in response to limited resources and time constraints. While we included all aspects of the patient handover, we specifically focus on infection care to demonstrate the benefits.

## Methods

We conducted the study in the adult medical ICU of a 950-bed tertiary public referral university hospital in Cape Town, South Africa. Nursing team patient handovers occur twice daily, at the end of day and night shifts. The existing handover standard operating procedure (SOP) was not designed for bedside use. We collaborated with clinical nurses, the unit nurse operational manager (OM), a nurse educator, and a quality improvement expert to co-design and pilot a nurse handover tool, with support from the ICU clinician lead and hospital nursing leadership. The OM facilitated collaboration, onboarding clinical nurses, and integrating research activities into the daily ICU operations. One researcher planned and co-ordinated the research activities, using graphic facilitation for nurse engagement in the co-design process. Regular check-ins and close partnerships between the researcher, quality improvement expert, OM, and nurse educator guided the process.

Using a participatory action research (PAR) framework and visual participatory methods, we adopted the plan-action-reflect strategy ^32^. The co-design of the ICU nurse handover tool progressed through three phases, outlined below.

### Baseline data collection of current handover practices and analysis

We conducted focussed ethnographic observation of nurse handovers to identify current practices. Our aim was to describe handover patterns between nurses at shift changes. Given our previous ethnographic observations for the wider PhD study, we used focussed ethnography, which allows for targeted observations within a limited period ^33– 35^. We used a previously developed data collection guide ^30, 36– 38^, and the researcher documented activities and interactions among nurses, focusing on the content and sequence of patient information exchanged. We thematically analysed ethnographic field notes using NVivo 12® software. QualCoder is a free and open source tool and could have been used as alternative software to aid qualitative data analysis. Open coding led to the development of a coding framework.

### Interactive nurse journal club using graphic facilitation

**Figure 1.**
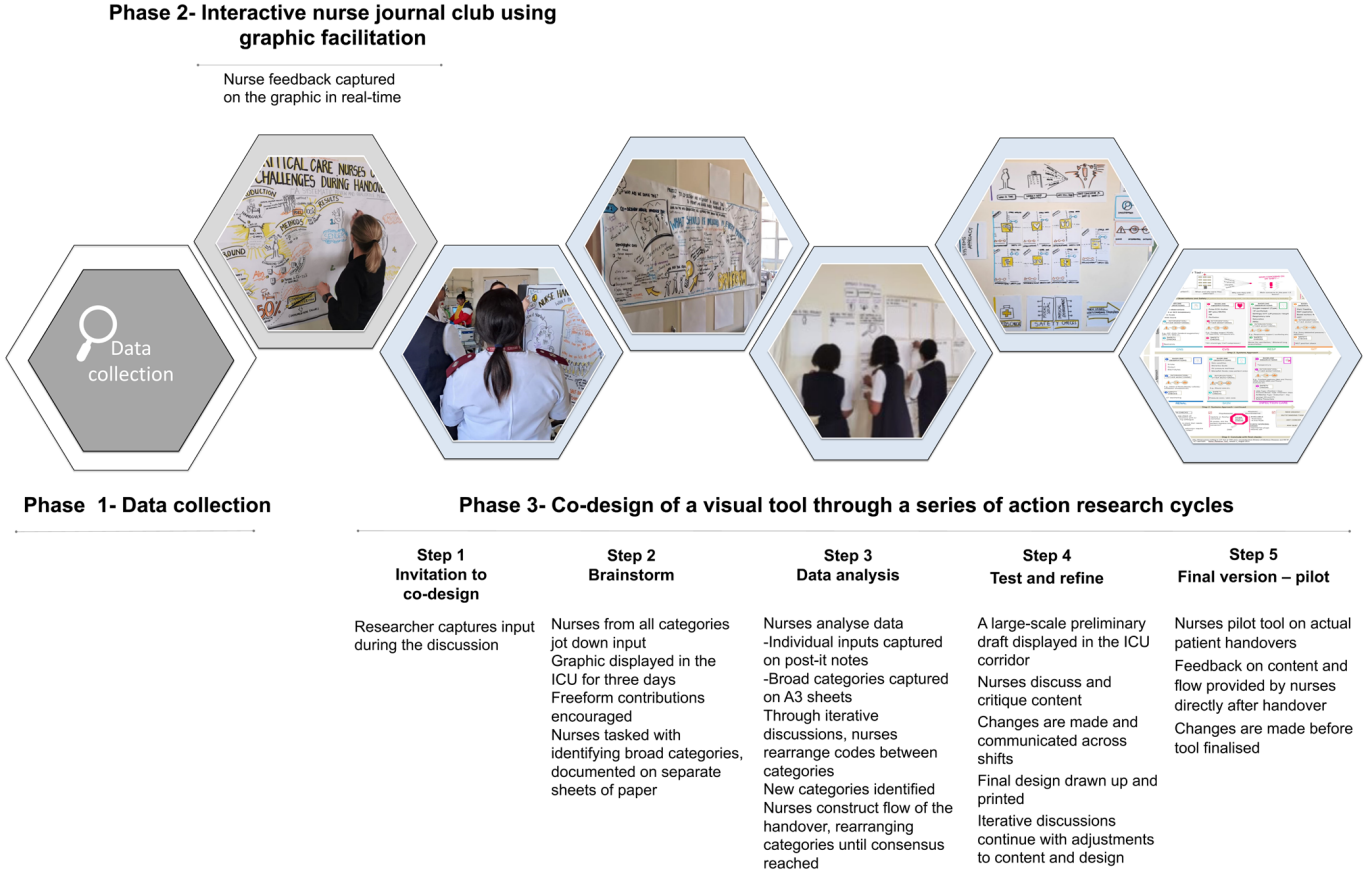
Collaborative graphic co-creation showing nurses' involvement in each phase.

Following Leonard and colleagues methodology for a participative journal club which used graphic facilitation to encourage participation ^39^, we held an interactive session with ICU nurses from the hospital. We chose to discuss a systematic review on communication challenges among critical care nurses and created a large-scale graphic outlining key discussion points. Nurses, organised into groups, discussed specific sections of the article and explored how it related to their handover challenges. The researcher recorded their feedback on the graphic during the group sessions, supporting the collaborative construction of the graphic. Figure 1 details nurse involvement in the graphic co-creation process during the phases of the project.

### Co-design of a visual tool through a series of action research cycles

***Step 1 ICU nurse feedback and invitation to co-design tool.*** Small groups of nurses gathered around a large hand-drawn graphic outlining the discussion. The objective was to discuss their handover experiences and challenges and to share insights on data the researcher collected from their own practices. After the researcher introduced the PAR process and the potential benefits of a visual map for handovers, nurses were invited to participate in the co-design. Nurse feedback was documented on the graphic and used across shifts to stimulate further discussion.

***Step 2 Brainstorm.*** Nurses contributed ideas to a large poster asking: 'What should be included in every handover?' This established a baseline of input and encouraged participation from nurses of various categories and shifts over a three-day period. The process resulted in diverse perspectives.

***Step 3 Self-analysis of recorded input.*** Nurses reviewed and analysed their collective input on the graphic using a deductive content analysis approach. This researcher facilitated this process, which followed an iterative plan-action-reflect strategy.

***Step 4 Test and refine draft.*** A large-scale draft of the handover map was displayed in the ICU corridor ( Figure 1). Nurses reviewed and refined the content and overall flow, identifying essential information for the tool. Changes to the tool were made and tested through a series of short iterative feedback and discussions across shifts, until consensus on the content and design was reached.

***Step 5 Pilot and refine the final version.*** The final tool was piloted over two weeks. The researcher assisted nurses during morning and evening rounds to use the tool, and gathered feedback. The tool was then presented to the Director of Nursing and the Nursing Board, who committed to its implementation and rollout in the unit and across other units in the Hospital.

### Ethical considerations

This study was part of a broader PhD study that explored team dynamics and communication to optimise clinical practice with a focus on the ICU multidisciplinary ward round. The University of Cape Town Human Research Ethics Committee (HREC) granted ethical approval for this study on the 1 September 2021, with annual renewals until study completion (HREC ref: 523/2021). Hospital approval was received on the 10 February 2022, after which informed consent was obtained from the ICU departmental head for the study to be conducted with the ICU staff. Subsequently, members of the ICU MDT, including nurses, were introduced to the broader study. Verbal consent was obtained from the participating ICU nurses after they were invited to participate in the co-design. As approved by HREC, verbal permission was required form direct observations of clinical practice. Thus, prior to the data collection episodes of nursing handover, nurses were reminded of the study’s purpose and offered an opt-out option, with no bearing on their clinical practice if they decided to withdraw.

## Results

Between September and October 2023, we conducted a pre-intervention qualitative analysis of 16 nursing handover ward round observations across 46 patient discussions over four hours. We then co-designed a pilot nursing handover tool through 18 contact sessions involving 31 nurses (April–June 2023) in an 8-bed medical ICU. Nurses from day and night shifts, representing various nursing categories and years of experience participated ( Table 1). First, we present the findings from ethnographic observations. Second, we explain how we engaged busy clinical nurses despite contextual limitations. Third, we highlight key insights from discussions that guided the content and lay-out of the visual tool.

**Table 1.** Nurse demographics in the co-design of a nursing handover sheet in the adult ICU.

Item	Biographical data	Count (n)	%
Gender	Male	4	13
	Female	27	87
Age	25-30 years	2	6
	31-34 years	10	32
	35-50 years	13	42
	Above 50 years	6	19
Shift	Day shift	15	48
	Night shift	16	52
Category	Professional nurse	14	45
	Enrolled nurse	9	29
	Enrolled nurse assistants	8	26
Qualification	Enrolled nurse assistants	8	26
	Enrolled nurse	9	29
	Diploma in nursing	6	19
	Bachelors in nursing	8	26
	Honours degree	1	3
	Master’s degree	1	3
Years of experience	<5 yrs 5–9 yrs	9 3	29 10
	10–19yrs	11	35
	20–29 yrs	3	10
	≥30 years	5	16

### General handover practices and specifics on infection care

Table 2 outlines general patterns in handover structure and content, including presentation sequence and detail levels (X1–4, Table 2). Reporting on the details and rationale of nurse-led activities and interventions, as well as the patients' clinical response to treatment/interventions, was inconsistent (X5–6, Table 2). The reference to charts (e.g. medication, observation and/or antibiotic charts) and devices such as infusion pumps to verify information, varied. (X7–8, Table 2). Staff shortages and unreliable public transport led to rushed handovers (X9–10, Table 2).

**Table 2.** The key emerging themes observed during nursing handover rounds.

Theme	ID	Quote
**Insights into general handover practices and specifics on infection care**	X1	The night nurse reports that the patient underwent a hysterectomy the previous day and is rebooked for theatre to remove swabs. "Now," she says, "her Glascow Coma Scale (GCS) has improved." She mentions that the Propofol was weaned overnight and that the patient is receiving additional boluses of Morphine. The central line is in position, and feeds were started via a nasogastric (NG) tube. She reports that she tested and confirmed that the NG tube is in position and mentions how much fluid she is aspirating every hour (in ml). Donning gloves, the day nurse adjusts and reduces the maintenance fluid and brings the rate down. After she removes the gloves, she sprays her hands. The night nurse unpins the medication charts, which include an antibiotic chart, and reads off what was administered overnight. She names the medications. Moving on, she reports that the skin is intact but says that the wound is oozing slightly.
	X2	The patient is on nasal prong oxygen and is asleep. The nurse begins by stating that there is nothing ‘special’ to report. She reports that the patient was on oxygen overnight and has remained stable. She then reports the temperature and, glancing up at the cardiac monitor, asks the day nurse how the day staff managed to monitor the patient's temperature every two hours. The day nurse asks for clarification, and the night nurse explains that the patient did not have a temperature probe in place. The day nurse admits uncertainty about how this was handled as she did not look after the patient. The night nurse continues, reporting that after she inserted the probe, the temperature was 38.2 degrees Celsius and subsequently decreased.
	X3	The nurse says, "There is nothing special here. The night was uneventful." She then proceeds to discuss the ventilator settings and reports that on the blood gas, the oxygen was low, but she reasons that it may have been an erroneous reading as it did not align with other indicators. She goes on to mention that his blood pressure was "fine and stable" and that the patient is communicating. She does not provide details on the form of communication as the patient is ventilated.
	X4	The nurse, who knows the patient well, mentions that the patient is a known diabetic and has been admitted to healthcare facilities on several occasions due to uncontrolled diabetes. She elaborates on the challenges over the past three days in maintaining stable glucose levels.
	X5	The nurse reports that they changed the arterial lines from the one side to the other but does not state the reason for the change.
	X6	The level and depth of information provided varies among nurses. Some offer detailed accounts, while others briefly mention issues without elaboration.
	X7	The nurse walked over to the charts, pulled the medication sheet off the board, and proceeded to list the medications given overnight. This list did not include any antibiotics.
	X8	The night nurse does not physically engage with the charts but mentions by memory the medications that were given overnight.
	X9	On arrival to the ward this morning, the night staff were sitting at the nurse's station, waiting. One nurse expressed her worry, mentioning that the day staff had not yet arrived and was concerned that she would miss her transport.
	X10	The group splits and some nurses have moved on to the next patient. The nurse who was in a hurry with regards to her transport does a brief handover of the other two patients.
	X11	The closing statement is that the temperature was a ‘little high’ but has improved this morning.
	X12	The nurse mentions the lines, positions. She goes on to report that feeds were started via a NG tube that’s position was confirmed.
	X13	Next, she describes how the arterial line trace was poor at around midnight. Although her intervention was to do a non-invasive blood pressure, the doctor had stated the importance of invasive monitoring due to his critical condition. With some repositioning, she managed to get the arterial line working.
	X14	The night nurse unpins the medication charts, which includes an antibiotic chart and reads off what was given overnight. She names the medications and does not add any additional information.

Temperature (‘apyrexial’/‘pyrexial’) was consistently communicated (X11, Table 2), but infection-related biomarkers such as white cell count (WCC) or C-reactive protein (CRP) and microbiological culture results were rarely mentioned. Invasive devices, particularly the positions and patency of invasive lines, were consistently included in the updates (X12-13 Table 2). Unless a line was removed, however, the duration of invasive devices and a prompt for line changes were not consistently reported.

Reference to antibiotic treatment generally depended on whether the handover nurse mentioned medications. Details on antibiotics ranged from stating that the patient is 'on antibiotics' to specifying the name. Antibiotic treatment details varied, with no consistent reporting on changes or de-escalation (X14, Table 2).

Nurses identified several gaps in handover practices during discussions, validating the observed data. They expressed frustration when lacking information from inadequate previous handovers, which impaired their ability to address patient-related questions during the MDT rounds.

### Leveraging visual methods and ‘7-minute scrums’ to optimise input from busy clinical nurses in the co-design process

Due to patient responsibilities and staff shortages, nurses could not be away from their clinical duties for long periods. We determined that nurses were more likely to fully engage in shorter, time-limited sessions held close to the clinical areas. We trialled and implemented brief, focused contact sessions, termed 'seven-minute scrums' to involve nurses.

These scrums proved valuable for brainstorming, especially with the large graphics displayed in the ICU. In later phases, the researcher placed the graphic in the corridor, inviting nurses to join facilitated discussions when their patient care duties permitted. The use of large-scale graphics in the unit, combined with recording nurses' input and discussions, and repeating the process with different shifts, facilitated an iterative process that built on the input from previous groups of nurses, while adhering to co-design principles. Table 3 summarises contextual challenges and innovative responses to optimise nurse engagement.

**Table 3.** Challenges, innovations, and key learnings in the co-design of the nursing handover tool.

Contextual and team challenges to consider in the co-design process	Adapting to the challenges: Innovations and solutions implemented
■ Nurses have limited time to attend meetings ■ In general, nurses often lack opportunities to participate in forums to discuss translating evidence into practice ■ Handover is a routine practice, with the associated challenges rarely discussed ■ Critically ill patients require continuous nursing supervision ■ Centralised budget cuts led to a reduction in nursing staff ■ An increase in workload for nurses and a suboptimal nurse-to-patient ratio ensues ■ Language and social dynamics between different nurse categories may impede active engagement	**Interactive journal club** ■ Engage large groups of nurses in a journal club session using graphic facilitation methods ■ This approach allows nurses from all categories to explore topics through generative discussions
	**Process** ■ Shorten nurse contact sessions to 7 minutes of focused discussions ■ Hold sessions in the ICU, ensuring that patients remain in direct view ■ Allow nurses the flexibility to leave the discussion to attend to patients ■ Use mobile graphics that can be wheeled into the ICU ■ Test for shared understanding of concept to overcome language barriers and enhance nurse engagement ■ Actively involve quieter nurses by encouraging their contributions ■ Invite nurses to share input outside of the scheduled group discussions ■ At the start of discussions, emphasise values such as collaborative teamwork, non-hierarchical structures, and equal appreciation of all contributions
Key learnings and insights gained from the co-design process	Implementing changes guided by key learnings and insights
**Interactive journal club** ■ Nurses discuss challenges they encounter during handovers ■ Nurses explore and discuss how the evidence from the article connects to their practical experiences ■ Feedback across groups highlights common challenges that are seldom discussed ■ Nurses contribute to the creation of a graphic, with their input recorded by the facilitator	
**Process** ■ Proximity to patients helps nurses stay focused on the discussion ■ A stand-up discussion around the graphic encourage higher levels of engagement and energy ■ In contrast, sit-down discussions (with nurses seated in and facing the graphic) leads to less input and lower participation ■ Setting time-limits for sessions allows nurses to organise patient work more effectively ■ Time pressure boosts engagement across all nurse categories and reduces hesitation in contributing ■ Spontaneous input, rather than over-thinking, is key to successful brainstorming ■ The researcher must have a clear session goal, thorough preparation, and skilled facilitation to optimise the 7-minute scrums	**Process** ■ Conduct most sessions in the ICU ward, using a 'stand-up discussion' approach ■ Keep contact sessions consistently limited to 7-minute scrums, even as staffing shortages improve ■ Schedule regular contact sessions but assess their feasibility daily based on the nurse-to-patient ratio and the severity of patients' conditions ■ The researcher must stay organised yet flexible to adapt to changing circumstances
**Tool** ■ Nurses debate whether information should be organised and reported by system or a task (e.g., grouping all observations together) ■ One nurse proposes a standardised approach for reporting interventions and investigations ■ Nurses acknowledge the importance of maintaining a logical flow of information in each section ■ Several revisions led to improvements in the key content to be included ■ Discussions reveal varied levels of understanding among nurses regarding routine practices (e.g., the frequency of nasogastric aspirates and how they are reported), highlighting practice gaps and opportunities for in-service education ■ Some aspects of the tool require nurses to report on details that may be beyond the immediate scope of knowledge, particularly for junior registered nurses and enrolled nurses	**Tool** ■ Adopt a body system approach for reporting system-related information. 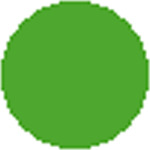 ■ Reach consensus and propose categorising body system-specific information into three sub-headings: Basic observation, Interventions/investigations, and Safety checks 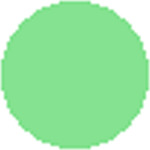 ■ Identify essential information for each section, aligning with the Hospital SOP and evidence based practices, and provide prompts 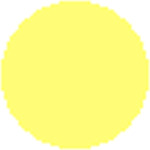 ■ Discussions lead to the innovative idea of incorporating a structured method for reporting intervention/investigation details, including the issue, actions taken, and resulting changes (if any) 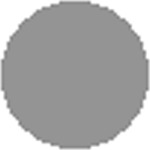 A standard icon is later developed to enhance this reporting method 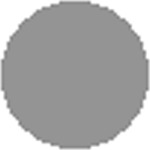 ■ Designate a separate reporting section for infection care 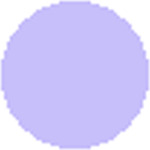 ■ Refine patient introduction and add a section for 'main concerns over the past 12 hours’ 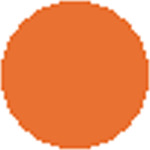 ■ Informed by experience and key patient information they want to receive when assuming patient care, nurses refine ‘final checks’ section 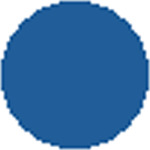 ■ Continuously iterate the design, lay-out and font size for further improvement
	* Coloured dots reference components of the Visual ICU nurse handover tool ( Figure 2)

### Co-design of the tool design and content

**Figure 2.**
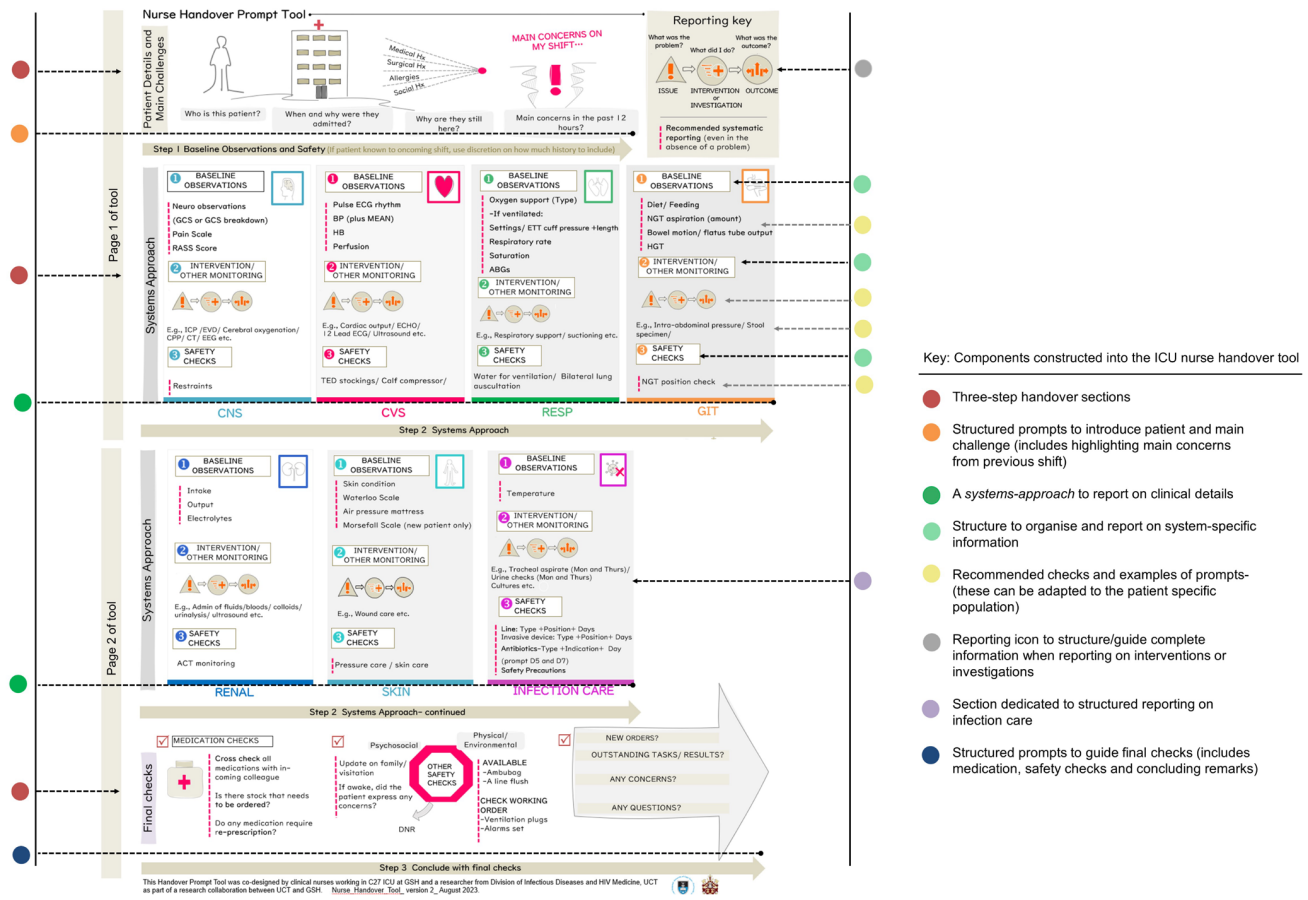
Visual ICU nurse handover tool and unique components constructed through the co-design process. This figure illustrates the final design of the two-page ICU nurse handover tool, which provides a structured approach to guide the handover process using icons and word prompts. It highlights the unique components incorporated into the tool through the co-design process.

***A body systems approach to report on clinical details.*** We adopted a body systems approach in line with the Hospital Handover SOP and evidence-based guidelines. The tool included three sections: a comprehensive patient introduction, a body systems overview, and final safety checks. Figure 2 illustrates the final tool, featuring the visual prompts for structured and detailed information.

***Organising body system specific information.*** Critical thinking discussions were guided by the questions: What is the problem? What is the intervention? How has the patient responded to the intervention? This approach helped to prioritise and organise system-specific patient information, for example information on infection care was typically spread throughout the handover. The temperature was reported alongside other observations, such as blood pressure, while the administration of antibiotics was mentioned with other medications. Nurses suggested a method for reporting system-specific monitoring and interventions, to improve inconsistencies identified in practice. For instance, details about a patient starting on inotropes varied-some nurses stated that the patient was on inotropes, while others included reasons for treatment and subsequent effects. To prevent infection care information from being overshadowed by other patient-related priorities and to ensure consistent reporting on IPC and AMS components, we added a separate infection care section to the tool. The infection section featured prompts for temperature and a reporting framework covering diagnostic tests, invasive devices, antibiotics, and infection-related safety precautions ( Figure 2).

## Discussion

We describe a nurse-led process to design a visual tool for structuring ICU nurse handovers in a LMIC setting. Using visual participatory methods, such as graphic facilitation, and engaging busy clinical nurses, we created a dynamic co-design process. Resource constraints necessitated innovative, short interactive sessions near the patients' bedside. Our participatory action research encouraged nurses to critically assess their current handover practices, to improve the existing process ^40^. The resulting output, a visually-based tool featuring a dedicated section on infection care, constitutes the distinctive aspects of our co-design approach.

Supportive leadership from managers and stakeholders is crucial for the successful outcome of an improvement project ^41^. The OM facilitated nurse attendance and served as a liaison between the researcher, clinical nurse, and nurse management teams. Regular check-ins between the researcher and the OM, nurse educator, and quality improvement expert ensured shared project goals and effective nurse engagement.

Effective co-design requires early collaboration with end-users to identify problems, develop context-specific solutions, and evaluate effectiveness ^42^. Nurses, as end users, are experts and crucial to the design process. Power inequalities commonly identified in co-design can lead to poor engagement and limited knowledge sharing, and a disparity between whose voice is heard and whose knowledge is valued ^43^. To account for issues related to hierarchies and power dynamics, fostering a supportive environment is essential, including multi-stakeholder engagement, trust building, respect for capabilities, and transparency ^15^. The researcher, a former nurse, participated as a collaborator, to balance power dynamics ^28^. Our objective was to ensure all nurses could equally contribute their ideas and experience to the construction of the content and design.

Visual methods, including various large-scale visual aids and facilitation, enhance participant engagement and foster a supportive process. The evidence suggests participants are more engaged when they see their input visually represented, promoting validation and acknowledgment ^26^.

The participatory co-design approach involves collaborating with participants without favouring one type of knowledge, promoting shared ownership ^15^. Interactive formats have proved beneficial for healthcare workers in research ^22, 23, 29^ and improvement initiatives ^28, 44^.

Graphic facilitation encouraged early nurse participation ^28^, shifting the focus from individual input to emerging collective group patterns. The researcher recorded feedback during the interactive sessions, creating a visual compendium of handover challenges, which were integrated into the graphic. Nurses were also encouraged to write their inputs on the graphic. To include nurses less likely to contribute in group discussions and ensure broad participation, while accounting for perceived power imbalances ^43, 45^, nurses were given an extended time to contribute their ideas individually. This approach helped to navigate issues related to the ‘dilemma of voice’ such as power dynamics, group size, and time constraints ^46^.

Short, time limited sessions in Phase 2 optimised nurse engagement by mimicking huddles-brief, focused, stand-up meetings that enhance information exchange between healthcare workers ^47– 49^. Huddles reduce hierarchical barriers, promote enhanced teamwork and safety culture, and improve patient care and clinical outcomes ^47, 48, 50^. Each co-design session had a clear goal, and maintaining focus and energy levels. Through these sessions, nurses across shifts saw their ideas evolve into the visual tool ^26^. Involving end users in the research and development process fosters a sense of ownership and buy-in, making them more likely to support and use the tool ^46^.

Visual communication, using visual elements to convey ideas, information, and data, is integral to daily life. Research in health education has shown that visual information enhances knowledge, understanding, and recall particularly when pictures are accompanied by associated words ^51^. Studies on nurses’ preferred learning styles indicate a strong preference for visual learning, with 75% of participants favouring this approach ^52^. Drawing from nurses’ tacit knowledge and experience, we integrated key components for nursing handover aligned with the SOP and evidence-based practices. These components were structured in an intuitive manner to mimic existing handover practices.

Our tool capitalises on the benefits of visual components by using simple icons and words to prompt crucial patient related information. It is designed to be user-friendly for nurses with various training and language backgrounds, prompting consistency and critical thinking in reporting patient interventions and documentation. This facilitated a comprehensive handover tool supporting safe patient care continuity between shifts. In Box 1, we summarise the unique features of the research process and visual tool design.

**Box 1. Unique features of the co-design process and the tool**

**Process**

**Exposure to research:** Nurses were introduced to elements of research and generative dialogue through (i) journal club and, (ii) analysis of content
**Leadership support:**Strong and consistent nursing and clinician support facilitate clinical nurse buy-in and nurse attendance and participation despite context-specific challenges. Leaders demonstrate an openness/ willingness to explore new approaches
**Visual methods enable participation by end-users**: Large scale graphic and visual participatory methods were used throughout to invite and facilitate active participation from all nurse categories, curbing perceived power imbalances and encouraging shared ownership and responsibility
**Use of limited time huddles**: Co-design by nurses, in an ICU ward setting using a 7-minute scrum approach

**Visual Tool**

Simple icons act as prompts
  Provides a standardised system and structured approach to guide the handover process
  Ensures no information is lost
  Report on system specific baseline observations, interventions, and safety checks
  Provides an approach to comprehensively report on interventions and investigations
  Includes structured information key to infection prevention and care
  Prompts can be adjusted to the patient specific population.

The need for a standardised system to guide nurse handover is well-documented ^1, 53– 55^. Our study showed inconsistencies in handover format and variations in the level of detail provided on clinical aspects, including infection-related information. As bedside practitioners crucial to patient continuity of care, nurses serve as key conduits for communicating key information to other healthcare workers ^13, 56, 57^. Structured communication enhances the accuracy and consistency of key points on AMS and IPC ^14^. In an ICU prioritising infection prevention and management, our focus was to incorporate key components of AMS and IPC into the nurse handover tool, reinforcing nurses' active roles.

## Limitation

Our study had several limitations. Observations were only conducted during the morning handover, potentially underrepresenting the evening shift. However, nurses rotated between day and night shifts, providing a sample of current practice. Clinical nurses from a single medical ICU participated in the study, limiting generalisability across the hospitals ten ICUs. Participatory approaches like graphic facilitation can introduce biases, as the nurse researcher may have unintentionally influenced the discussion. To mitigate this, regular check-ins were scheduled with the quality improvement expert and OM to assess the process direction and development. The 7-minute scrums may have limited the depth and quality of contributions. While tested with other nursing groups who agreed on the key components included in the tool, they suggested adapting the prompts for different patient populations. The tool can be adjusted for various clinical settings with appropriate language and icons. The Hospital Quality Improvement Group has adopted the tool for implementation and measurement of its impact on safety outcomes in this unit and other hospital units.

## Conclusion

Co-design practices in resource-limited settings are emerging, with limited application in health research. Our study describes a co-designed, quality improvement process that actively engaged nurses in designing and adopting a visual, structured handover tool in a low-resource setting. This process counters some of the co-design challenges such as resource scarcity, and difficulties involving those with less agency and power in the healthcare team dynamic. The visual tool uses icons and word prompts to systematically report patient care risk factors, body systems, IPC and AMS. This study demonstrates the potential for improving clinical practice through collaborative efforts between research and clinical teams, offering a unique, adaptable handover tool that can be tailored to local context and patient needs.

## Ethics and consent

The University of Cape Town Human Research Ethics Committee (HREC) granted ethical approval for this study on the 1 September 2021, with annual renewals until the study’s completion (HREC ref: 523/2021). Hospital approval was received on the 10 February 2022, after which informed consent was obtained from the ICU departmental head for the study to be conducted with the ICU staff. Subsequently, members of the ICU multidisciplinary team (MDT), including nurses, were introduced to the broader study. Verbal consent was obtained from the participating ICU nurses after they were invited to participate in the co-design. As approved by HREC, verbal permission was required form direct observations of clinical practice. Thus, prior to the data collection episodes of nursing handover, nurses were reminded of the study’s purpose and offered an opt-out option, with no bearing on their clinical practice if they decided to withdraw.

## Data Availability

ZivaHub: Open Data UCT: Ethnographic observations from Nursing Handovers in ICU. https://doi.org/10.25375/uct.26819308 ^58^. This project contains the following underlying data: Final_Nurse Observations_ ethnographic.docx Data are available under the terms of the Creative Commons Attribution 4.0 International license (CC-BY 4.0).
