# Supplementary material for: Co-design of a nurse handover tool to optimise infection control and antimicrobial stewardship in a low resource setting intensive care unit: A nurse led collaboration
Source: Wellcome Open Res. 2024 Oct 15;9:583. [Version 1] doi: 10.12688/wellcomeopenres.22931.1 (PMC11803200; doi:10.12688/wellcomeopenres.22931.1)
Supplement: Peer review history

# Reviewer report: Reviewer response for version 1

**DOI:** 10.21956/wellcomeopenres.25249.r113240

**Published:** 2024-12-30

**Authors:** Karin A Thursky

**Affiliations:** 1 Health Services Research, Peter MacCallum Cancer Centre (Ringgold ID: 3085), Melbourne, Victoria, Australia; 2 The Guidance Group, The Royal Melbourne Hospital (Ringgold ID: 90134), Parkville, Victoria, Australia; 3 National Centre for Antimicrobial Stewardship, Department of Infectious Diseases, The University of Melbourne (Ringgold ID: 2281), Melbourne, Victoria, Australia

I note the comments from Reviewer 1 and 2 about having post implementation results available. However, the opportunity to present the full co-design process is often limited. This is important research which demonstrates the hands-on approach to the co-design of an intervention in a high pressure and challenging environment. The study team should be congratulated on this innovative program.

**The article could be improved with some additional considerations:**

- Was the intent to explicitly establish the tool as a bed-side handover? This is not confirmed although there was a comment in the methods about the fact that existing handover standard. This point needs clarification.
- There is a lot of rich information in the ethnography from the supplement. I believe that Table 2 could be enriched with thematic grouping elements and more details and quotes. It is not clear in the Table what an ID is (is this a person), do the comments relate to a single patient or multiple patients? 
- The images in Figure 1 are very interesting and should be made available in the supplement given that the graphic representation was highlighted as a novel element of this program.
- Is the tool something that is printed as PDF? What form is it produced? Were there guidelines on how to write notes for the handover based on this tool. A bit more about the planned implementation and next steps would be helpful.
- I am not sure it is necessary to refer to the PhD program in the paper, as this should be standalone document. 

Is the work clearly and accurately presented and does it cite the current literature?

Yes

If applicable, is the statistical analysis and its interpretation appropriate?

Yes

Are all the source data underlying the results available to ensure full reproducibility?

Partly

Is the study design appropriate and is the work technically sound?

Yes

Are the conclusions drawn adequately supported by the results?

Yes

Are sufficient details of methods and analysis provided to allow replication by others?

Partly

Reviewer Expertise:

Antimicrobial stewardship, infectious diseases, health services research

I confirm that I have read this submission and believe that I have an appropriate level of expertise to confirm that it is of an acceptable scientific standard, however I have significant reservations, as outlined above.

# Response

**Published:** 2025-01-12

**Authors:** Candice Bonaconsa

**Affiliations:** University of Cape Town Department of Medicine, Observatory, Western Cape, South Africa

Dear Professor Thursky,

 The authors sincerely thank you for your review and for highlighting the importance of a hands-on approach to co-designing an intervention in a high-pressure environment.  We value the specific and constructive feedback you provided, which we will carefully consider and address in a point-by-point format alongside the input from other reviewers. Thank you for the opportunity to enhance our manuscript further. We look forward to resubmitting the revised version for your consideration.

 Kind regards, 

 Candice Bonacsona,

 on behalf of the co-authors

# Reviewer report: Reviewer response for version 1

**DOI:** 10.21956/wellcomeopenres.25249.r106072

**Published:** 2024-12-30

**Authors:** Nour Shamas, Fatima Aldawood

**Affiliations:** 1 Independent antimicrobial stewardship consultant, Beirut, Lebanon; 2 Department of Infection Prevention and Control, King Abdulaziz Medical City (Ringgold ID: 535431), Riyadh, Riyadh Province, Saudi Arabia

This study describes the process of co-developing a nursing handover tool with an initial focus on infection prevention and control (IPC) and antimicrobial stewardship (AMS). Overall, this paper describes an excellent collaborative effort, the impact of which has yet to be evaluated. As the work is collaborative and multidisciplinary, so is this review with a nurse specialized in pediatric critical care, infection control, and antimicrobial stewardship and a clinical pharmacist specialized in infectious disease and antimicrobial stewardship as co-reviewers.

**The study presents various strengths:**

- The authors used an interesting approach which uses qualitative research to develop a practical bedside tool that improves patient care in general including infection prevention and control and stewardship.
- The tool is developed by nurses and their colleagues for nurses through a collaborative approach potentially leading to increased interest and uptake of a practical innovative tool.
- Table 3 is very insightful, especially the adaptation of duration and location of the discussions to the needs of participating nurses rather than asking them to adjust to a new research process.
- Efforts to tailor the methods to involve quieter nurses is very well received as well as the overall resource efficient approach of the study.
- It is commendable that the authors mentioned an alternative open-source qualitative data analysis software, suitable for any setting, which can facilitate the replication of the work.
- Box 1 is very insightful and helps promote replication of the research efforts elsewhere.

**A few things could be improved:**

- The main unique feature of the tool is using a visual design which the authors could consider reflecting in the title.
- Although the initial interest of this study may have been to focus on IPC and AMS, the developed tool is multidimensional and the IPC and AMS portions are just one portion of the tool. It would be interesting to verify and highlight this in the in introduction and throughout as this multidimensional approach adds even more value to the developed tool.
- The authors use the terms “infection prevention”, “infection care”, “infection management” throughout the document, potentially interchangeably - each of which could be understood differently. It could be useful to define or unify the terms used depending on if the aim was to use them interchangeably or if there is a clear difference in scope. “Infection care” as we understand it for example, wouldn’t usually include infection prevention and control and starts from when the infection occurs, and doesn’t necessarily include stewardship principles.
- It would be interesting to know more about what led to the development of this work. The authors mentioned that “the existing handover standard operating procedure (SOP) was not designed for bedside use” but does not elaborate further to identify what was considered lacking and how this was determined, except for a short paragraph at the end of the discussion. The addition of a few lines on this could help to others in understanding the rationale for the study and can help support others in replicating the process.
- No pharmacists (or clinical pharmacists) were mentioned to be part of the multidisciplinary team involved in the development of the tool although that would be an interesting addition in the future if available in that setting.
- Did the journal club process evolve based on the key learnings and insights gained from the interactive sessions? Were the nurses encouraged to read the article beforehand? This is particularly significant as involving nurses in journal clubs, while highly valuable, is not a common practice in some regions. Additional details about the authors’ experiences and methodology would provide valuable insights.
- The authors may consider adding the reference for the article used in the journal club in the initial section under “interactive nurse journal club using graphic facilitation”
- In Figure 1, the comments pertain to phase 3, but this isn’t referenced in the text. It might be helpful to include this information alongside the title: "Co-design of a visual tool through a series of action research cycles." Additionally, the authors may consider whether anything could be added for phases 1 and 2 without making the text redundant.
- Providing brief definitions or graphic representations to the approaches used such as the plan-action-reflect strategy and the deductive content analysis approach would be valuable in facilitating the reproduction of this work. This is especially important since many stakeholders in the fields of nursing, antimicrobial stewardship, infection control, pharmacy, infectious disease, and others may not be familiar with these concepts, which could impede replication and implementation.
- Further information on how long each step took is interesting. For example, for the following:
   
  - “Changes to the tool were made and tested through a series of short iterative feedback and discussions across shifts, until consensus on the content and design was reached.”
  - The authors say that the discussion took 4 hours. It would be good to clarify further if this is due to several 7-minute sessions added all together or if this is not the case. This again could all be clarified with more information on the process and the approximate time each step took.
- Minor typos to be fixed:
   
  - “form” instead of “from” in line 8 under Ethical considerations – and the sentence “verbal permission was required form direct observations of clinical practice” is not very clear.
  - The concept of a “body system” was not directly clear and I initially thought it was a buddy system to improve communication between nurses. The authors may consider elaboration. 
- Figure 2 is not clear and it is highly advisable to improve this.
- Could the authors clarify if the work was done solely on antibiotics or if it also includes antifungals and antivirals, which are also very important?
- It is not clear if the handover tool also addresses infection precautions (standard, contact, or respiratory...) or therapeutic drug monitoring, both of which are important aspect of the nurses’ role in infection control and antimicrobial stewardship, respectively.
- There are a few short paragraphs (paragraphs 4 and 5 of the discussion) that could be edited to improve flow.
- In the discussion, it is mentioned that including AMS and IPC components in the handover tool reinforce nurses’ active roles. Highlighting some practical examples of AMS and IPC elements would serve as a guide to what can be included.

We found this work very interesting and look forward to seeing more on the satisfaction of nurses and medical teams using this visual tool as well as the impact of the tool’s implementation on patients in different patient care areas in future publications.

Is the work clearly and accurately presented and does it cite the current literature?

Yes

If applicable, is the statistical analysis and its interpretation appropriate?

Yes

Are all the source data underlying the results available to ensure full reproducibility?

Yes

Is the study design appropriate and is the work technically sound?

Yes

Are the conclusions drawn adequately supported by the results?

Yes

Are sufficient details of methods and analysis provided to allow replication by others?

Yes

Reviewer Expertise:

Infectious diseases, antimicrobial stewardship practice and policy, clinical pharmacy

We confirm that we have read this submission and believe that we have an appropriate level of expertise to confirm that it is of an acceptable scientific standard.

# Response

**Published:** 2025-01-12

**Authors:** Candice Bonaconsa

**Affiliations:** University of Cape Town Department of Medicine, Observatory, Western Cape, South Africa

Dear Dr. Shamas,

 The authors sincerely thank you for your valuable input to refine and improve this manuscript. We particularly appreciate the diverse and collaborative expertise brought to the review, including perspectives from a paediatric critical care nurse specialist and a clinical pharmacist. Your clear and constructive feedback is highly valued, and we will carefully address your points in a detailed, point-by-point format alongside the input from other reviewers. Thank you for the opportunity to strengthen our manuscript. We look forward to resubmitting the revised version for your consideration.

 Kind regards,

 Candice Bonaconsa,

 on behalf of the co-authors

# Reviewer report: Reviewer response for version 1

**DOI:** 10.21956/wellcomeopenres.25249.r114031

**Published:** 2024-12-11

**Authors:** Nickcy Mbuthia

**Affiliations:** 1 Department of Medical Surgical Nursing, Kenyatta University, Nairobi, Nairobi County, Kenya

This research article discusses the co-design of a visual, structured nurse handover tool aimed at optimizing infection control and antimicrobial stewardship in a low-resource intensive care unit in South Africa. The study actively engaged nurses in the development process using a participatory action research framework with visual participatory methods. This timely quality improvement project outlines a replicable approach for similar settings. However, the title and certain sections of the ethical considerations suggest a multidisciplinary collaboration, while the process was exclusively nurse-focused, with no involvement of other healthcare workers. Additionally, the authors mention that the tool was pilot-tested and subsequently implemented in other departments. However, they do not present the pilot results or detail the revisions made to the tool following the pilot phase. It would greatly improve the paper, especially showing how it optimised infection prevention and antimicrobial stewardship as this is the main focus of the project.

**Further improvements that they can make to improve the paper include:**

- Under the “Interactive nurse journal club using graphic facilitation” provide a citation for the systemic review article that they used to facilitate the discussions.
- Provide timelines for the activities in the methods section (not the results) and make sure they are consistent with what they provide in the abstract.
- The descriptions of the activities in the phases are best provided in the methods section and not the results.
- Provide information on the strategies they employed to maintain the rigor of the study including the ethnographic observations of the handovers, discussions etc.
- Breakdown the themes that have been detailed in the results sections to make Table 2 more understandable.
- The main objective was to develop a handover tool that optimises infection prevention and antimicrobial stewardship. In the discussions with the nurses, the authors would have brought out the challenges regarding the handover of these 2 elements explicitly. Additionally, the codesign would have shown how they optimised these 2 elements in the tool.
   
  - Moreover, the discussion (which is more skewed to a discussion about the codesign process) would have also included a discussion of how the tool achieved the objective.

Is the work clearly and accurately presented and does it cite the current literature?

Yes

If applicable, is the statistical analysis and its interpretation appropriate?

Not applicable

Are all the source data underlying the results available to ensure full reproducibility?

Yes

Is the study design appropriate and is the work technically sound?

Yes

Are the conclusions drawn adequately supported by the results?

Yes

Are sufficient details of methods and analysis provided to allow replication by others?

Yes

Reviewer Expertise:

Critical care nursing, Patient Safety

I confirm that I have read this submission and believe that I have an appropriate level of expertise to confirm that it is of an acceptable scientific standard, however I have significant reservations, as outlined above.

# Response

**Published:** 2024-12-17

**Authors:** Candice Bonaconsa

**Affiliations:** University of Cape Town Department of Medicine, Observatory, Western Cape, South Africa

Dear Dr. Mbuthia,

 The authors wish to thank you for your thoughtful review and the opportunity to improve our manuscript. We are encouraged that you view this as a timely and replicable quality improvement project. We are especially grateful for the specific and constructive feedback you have provided. These, along with the comments from Reviewer 1, will be carefully addressed in a point-by-point format. We will then resubmit the revised manuscript for further review.

 Thank you once again for your clear and considered pointers to guide us.

 Kind regards,

 Candice Bonaconsa, on behalf of the co-authors

# Reviewer report: Reviewer response for version 1

**DOI:** 10.21956/wellcomeopenres.25249.r107927

**Published:** 2024-11-15

**Authors:** Jayanta B Sarma

**Affiliations:** 1 Northumbria Healthcare NHS Foundation Trust, North Shields, England, UK; 2 Microbiology, Letterkenny University Hospital (Ringgold ID: 57987), Letterkenny, County Donegal, Ireland

The research outlines an approach to and describes the process of designing an ICU handover tool for nurses in a resource-limited country. The objective is to remedy the prevailing communication deficiency and ensure that all critical elements, such as Infection Control and Anti-Microbial Stewardship, are consistently mentioned. The development process was participatory and based on an established framework. I find the effort very interesting and believe this could be replicated elsewhere. However, the study did not go to its logical conclusion. How successfully the tool is being used, and its impact on clinical outcomes such as lower ICU stay or improved antimicrobial prescribing such as appropriate duration of treatment and similar other parameters are not addressed/reported in the study. The results from the post-implementation phase are required to complete the loop of this study, which is described as a quality improvement initiative.

Is the work clearly and accurately presented and does it cite the current literature?

Yes

If applicable, is the statistical analysis and its interpretation appropriate?

Not applicable

Are all the source data underlying the results available to ensure full reproducibility?

Yes

Is the study design appropriate and is the work technically sound?

Yes

Are the conclusions drawn adequately supported by the results?

Yes

Are sufficient details of methods and analysis provided to allow replication by others?

Yes

Reviewer Expertise:

Infection control, anti-microbial stewardship, hospital-acquired infection.

I confirm that I have read this submission and believe that I have an appropriate level of expertise to confirm that it is of an acceptable scientific standard.

# Response

**Published:** 2024-12-18

**Authors:** Candice Bonaconsa

**Affiliations:** University of Cape Town Department of Medicine, Observatory, Western Cape, South Africa

Dear Dr. Sarma,

 The authors wish to thank you for reviewing our manuscript.

 We are encouraged that you found the work interesting, particularly your observation that the participatory process we described has the potential to be replicable. This underscores what we were trying to achieve in this study. We appreciate your insightful feedback regarding the gap that we have not measured the successful use or impact of this approach on clinical outcomes.

 We recognize the value of incorporating results from a post-implementation phase to complete the loop of this quality improvement initiative. While the purpose of this paper was to describe the co-design process—since implementation is still ongoing—your comments have helped us to work on better clarifying the study's current limitations. The changes recommended by yourself and Reviewer 2 will be carefully addressed in a point-by-point format, and we will resubmit the revised manuscript for further review.

 Thank you once again for your assistance in helping us improve this manuscript.

 Kind regards,

 Candice Bonaconsa, on behalf of the co-authors
